# Why does *Daphne pseudomezereum* drop its leaves in the summer? An adaptive alternative to surviving forest shade

**DOI:** 10.1111/ppl.12972

**Published:** 2019-05-07

**Authors:** Thomas Lei, Naoko Yamashita, Takuya Watanabe, Takayuki Kawahara, Tomiyasu Miyaura

**Affiliations:** ^1^ Department of Environmental Solution Technology Ryukoku University Otsu 520‐2194 Japan; ^2^ Forest Ecology Group, Kansai Research Centre Forestry and Forest Products Research Institute Kyoto 612‐0855 Japan; ^3^ Hokkaido Research Center Forestry and Forest Products Research Institute Sapporo 062‐8516 Japan

## Abstract

*Daphne pseudomezereum* A. Gray (Dpm) appears to be the only woody species in the north temperate forest that sheds its leaves in the summer while remaining green over winter (i.e. wintergreen leaf habit). Yet, the reason for this odd leaf habit has not been explored. To this end, we examined the microclimatic settings and ecophysiological traits of Dpm and its three native congeners in a field study of eight natural populations. In addition, we conducted a common garden experiment using Dpm plants where potential carbon gain across the seasons was estimated, using actual field microclimate data. Together, these data tested the hypothesis that Dpm retained traits of an open‐grown upland ancestor, unable to adapt to the deep summer shade, it survived by becoming summer dormant and wintergreen. Our hypothesis was supported by patterns of leaf ecophysiological traits and carbon gain simulations in Dpm, consistent with the energetic feasibility of a summer dormancy followed by an autumn leaf sprout. We also conclude that carbon deficit driven by low light and high respiration cost is the trigger for the leaf habit of Dpm and assert that its phenological strategy represents a rare but viable alternative strategy for persistence in the temperate understory.

**Abbreviations**
LCP: light compensation point
LMA: leaf mass per area ratio
PAR: photosynthetically‐active radiation
PPFD: photosynthetic photon flux density
Rd: respiration rate

## Introduction

Given the primary function of a leaf as a photosynthetic organ, it is not surprising that carbon gain and leaf life span are intimately related (Chabot and Hicks 1982). Not only is the timing and duration of leaf presence important to whole plant fitness (e.g. Kikuzawa and Lechowicz 2011), their variations in a plant community can influence the invasive success of some species (Fridley 2012) and impact forest dynamics in current and future climates (Kikuzawa et al. 2013, Reich et al. 2016). There has been much discussion about the functional basis of leaf longevity and leaf habits, as well as the driver for the observed distribution of deciduous and evergreen species (Chabot and Hicks 1982, Kikuzawa 1991, Reich et al. 1992, Givnish 2002, Wright et al. 2004, Kikuzawa and Lechowicz 2011). However, among the existing types of leaf habits, *Daphne psudomezereum* (together with its northern relative, *Daphne jezoensis* syn. *Daphne kamtschatica*) stands out as an exception by being a summer deciduous shrub in the temperate forest (Kikuzawa 1984, Lei and Koike 1998).

**Figure 1:**
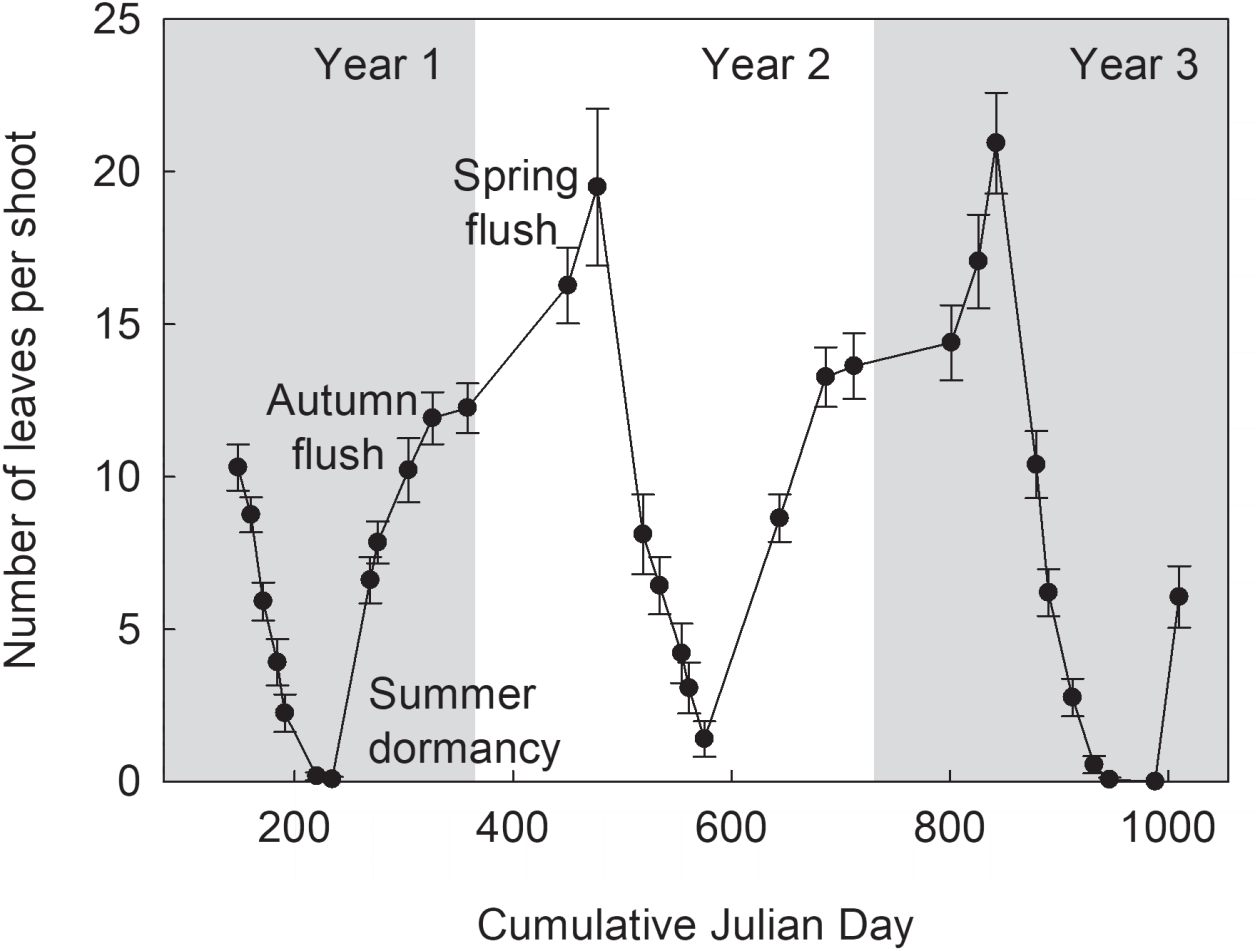
Leaf phenology of *Daphne pseudomezereum* population at the Ibu site (Table 1). Tagged shoots were monitored over complete 2 years of growth (including three winters). The number of leaves per shoot is derived from the mean of 13 shoots ± se, at one shoot per plant.

*Daphne pseudomezereum* (Dpm), belonging to the family of Thymelaeaceae, is a small, ‘deciduous’ woody shrub native to Japan. The genus *Daphne* is a group of mostly forest understory shrubs with 92 accepted species (http://theplantlist.org) distributed from the temperate regions of Europe to Asia, extending south to tropical Southeast Asia, Polynesia and Australia. Unlike any other co‐occurring plants in the temperate forest, Dpm and *D. jezoensis* (determined to be the southern and northern races, respectively, of a single species based on molecular phylogeny; T. Kawakara unpublished data) undergo summer senescence under dense canopy shade. This is followed by a flush of leaves in late summer that remain green over winter when the predominantly deciduous canopy is open (Table 1, Fig. 1; images of the *Daphne* species are provided as Supporting Information). An additional leaf flush occurs in the spring, in sync with many winter dormant species, but both autumn and spring leaves are shed between July and September. This leaf habit is commonly known as wintergreen (Bell and Bliss 1977). Our study is an attempt to shed light on the reason for this unusual leaf phenology, which appears to be a shade‐avoidance mechanism, from an ecophysiological and evolutionary perspective.

**Table 1:** Location, leaf habit and forest environment of the four *Daphne* species in this study. WG, wintergreen; SG, summer green; EG, evergreen. Forest type includes dominant overcanopy species present. Species name are based on accepted nomenclature (or alternatively, synonyms with high confidence level) given in The Plant List http://theplantlist.org (accessed 2019/1/28).

Species	Leaf habit	Location code	Location	Latitude/Longitude (elevation in m)	Forest type and dominant species (deciduous species in bold)
*Daphne pseudomezereum* A. Gray	WG	Ibu	Mt. Ibuki, Shiga Pref.	35°23′57″N, 136°23′16″E (472)	Mixed broadleaf forest, ***Carpinus laxiflora***, ***Quercus mongolica subsp. crispula***, ***Q. variabilis***
		Ryo	Mt. Ryozen, Shiga Pref.	35°17′4″N, 136°21′53″E (651)	Mixed broadleaf forest, ***Cornus controversa***, ***Zelkova serrata***
*Daphne koreana* Nakai	SG	Gyo	Gyojagaeri, Nara Pref.	34°11′38″N, 135°56′39″E (1449)	limestone outcrop, scattered trees, *Chamaecyparis obtusa, **Fagus crenata***
		Tsu	Mt. Tsurugi, Tokushima Pref.	33°51′38″N, 134°05′40″E (1728)	limestone outcrop, low trees and shrubs, *Abies veitchii, **Betula ermanii***
*Daphne kiusiana* Miq.	EG	Kib	Kurama, Kyoto Pref.	35°07′46N, 135°46′8″E (526)	*Chamaecyparis obtusa* plantation forest
		Nag	Mt. Nagasaka, Hyogo Pref.	34°44′39N, 135°07′27″E (385)	*Cryptomeria japonica* plantation forest
*Daphne miyabeana* Makino	EG	Mik	Mikuni, Kyoto Pref.	35° 19′16″N, 135°47′42″E (929)	Mixed broadleaf forest, ***Carpinus laxiflora***
		Hin	Mt. Ryuo, Shiga Pref.	35°01′24″N, 136°19′22″E (787)	Mixed broadleaf forest, ***Quercus mongolica subsp. crispula***

To understand the unusual phenological behavior of Dpm, we begin with the classical interpretation of variations in leaf phenology as the consequence of the environment, affecting how leaves accrue their investment cost and how much each leaf contributes to whole plant carbon gain (Monk 1966, Chapin III 1980, Chabot and Hicks 1982, Givnish 2002, Kikuzawa and Lechowicz 2011). Research has shown that a leaf can recover its cost of investment in as little as 22 days after leaf emergence under high light and 43 days in partial shade (*Fragaria* species, Jurik and Chabot 1986). Having accrued the initial leaf cost, the leaf can accumulate carbon profit, minus the maintenance cost mainly as respiration). But depending on the speed of decline in assimilative capacity (faster under high light), a quicker leaf turnover (i.e. shorter leaf lifespan) may be more profitable for the plant (Jurik and Chabot 1986, Kikuzawa 1991, Xu et al. 2017). The longer payback time for shade leaves necessitates longer leaf lifespan and because their photosynthetic performance declines more gradually, leaf longevity can extend beyond the payback time and lead to more carbon benefit for shade leaves (Jurik and Chabot 1986). It is therefore reasonable to expect individual leaf longevity to be determined by its intrinsic carbon economy and physiological status with additional regulation imposed by unfavorable seasonal conditions, such as cold or drought on the whole plant leaf habit.

Relative to a more ‘disposable’ deciduous leaf, evergreen leaves are generally higher in leaf mass per area ratio (LMA) and tend to occur in more shaded environments. Therefore the time needed to recover their leaf construction cost is longer, resulting in longer leaf lifespans (Wright et al. 2004, Valladares and Niinemets 2008). During their longer payback period, evergreen leaves often experience additional stresses such as drought and cold that limit carbon gain, but they may also benefit from seasonal improvements in light, during open winter canopies in many temperate forests (Miyazawa and Kikuzawa 2005). Deciduous shrubs and tree seedlings with early season bud‐burst (Seiwa 1999, Augsperger et al. 2005) adopt a similar strategy for carbon capture and delay leaf senescence past the ‘post‐canopy’ period (Fridley 2012). For Dpm, by flushing leaves in the autumn and retaining them over winter, carbon gain in both post‐canopy and early spring pre‐canopy periods can be achieved. But paradoxically, by doing so, the cohort of ‘deciduous’ leaves must survive the cold winter months that would normally have triggered leaf fall.

When delving into leaf phenology, a clear distinction between individual leaf lifespan and whole plant leaf habit must be made, because they respond differently to intrinsic demands and environmental constraints. Regardless of individual leaf lifespan, which can range between a few weeks to decades (Wright et al. 2004, Kikuzawa and Lechowicz 2006), leaf lifespan by itself does not define leaf habit. Only when lifespan of all leaves is reached synchronously on the plant during a 12‐month period does the deciduous habit arise (Chabot and Hicks 1982). Some plants produce only leaves with longevity less than 1‐year, but because they senesce asynchronously, the tree remains evergreen (Kikuzawa and Lechowicz 2006). Independent of the complex regulation involved in individual leaf senescence (which defines leaf longevity; Woo et al. 2013), the synchronous leaf shedding (i.e. leaf habit) would further require a systemic and coordinated whole‐plant response. While the precise factors that trigger a deciduous leaf habit are not known, it is assumed that the onset of plant level carbon deficit, be it winter freeze or seasonal drought that is likely to prevail over a significant period, plays a significant role in initiating the process. However, instead of freezing or drought, the coordinated leaf shedding of Dpm appears to concur with low light and high temperature occurring in mid‐summer. This seems to be unique among forest understory species, because they commonly persist through summer by physiological and morphological means (Valladares and Niinemets 2008). This leads to the hypothesis that Dpm lacks sufficient physiological shade adaptation, and instead, has evolved an alternative (summer deciduous) strategy to avoid this period of environmental stress.

To test this hypothesis, we examined key ecophysiological traits of Dpm and assessed whether summer dormancy is related to their lack of leaf shade adaptation. To put the leaf properties of Dpm in context, we compared it with three of its evergreen and summergreen congeners to highlight its phenological anomaly as part of an alternative functional design. Congeners include two evergreens, *Daphne miyabeana* and *Daphne kiusiana*, differing in their preference for mixed forests and evergreen conifer forests, respectively, and the summergreen *D. koreana* *,* occurring mainly on limestone outcrops greater than 1000 m in elevation (Table 1). *Daphne koreana*, while sharing a particularly close genetic affinity with Dpm (T. Kawahara, unpublished), exhibits a diametrically opposite leaf phenology. Based on both a field study of the four species and growth chamber simulation experiments, we found evidence supporting Dpm's unique leaf phenology as a viable adaptive strategy (Marks and Lechowicz 2006).

## Materials and methods

### Study site

Within the naturally occurring populations of the four species of *Daphne* in western Japan, two populations per species were selected for this study. Their locations and the prevailing forest type and common canopy tree species are given in Table 1.

### Environmental attributes of the *Daphne* habitat

At each location, we assessed the long‐term microclimate changes in irradiance, air temperature, soil temperature and soil moisture by using data loggers and sensors from Onset Computer Corp, (Borne, MA). The sensors used for irradiance were Hobo PAR smart sensors mounted on posts between 0.5 and 1 m above ground (adjusted to just above the canopy height of adjacent *Daphne*); for air temperature, Tidbit v2 Temp Loggers were mounted at ca. 0.8 m aboveground; for soil temperature, 12‐Bit Temp Smart Sensors were inserted belowground at a depth of ca. 15 cm and for soil moisture, Soil Moisture Smart Sensors (20 cm‐long probe) were positioned vertically from ca. 3 cm below the soil surface. The sensors (excluding the Tidbit v2 Temp Loggers) were connected to the Hobo Micro Station Data Loggers. To obtain an average condition of the location, three complete sets of sensors were placed at each site roughly 10 m apart. Sensor readings taken at 2 min intervals were continuously recorded and maintained for at least 2 years. In addition to the photosynthetically‐active radiation (PAR) sensor, forest light was also assessed using canopy photographs. These were taken with a Nikon Coolpix 990 digital camera and a Nikon Fisheye Converter FC‐E8 0.21× lens with the camera lens placed at the canopy height of *Daphne*. Between 17 and 32 images were taken at each location under overcast sky. The images were analyzed using CanopOn2 by A. Takenaka (http://takenaka-akio.org/etc/canopon2/, in Japanese). The software (presently with instructions in Japanese only) was developed based on Anderson (1964) with minor modifications (A. Takenaka, pers. comm.). Canopy openness (%) was derived from the image analysis.

### Ecophysiological attributes of *Daphne*

To assess the carbon assimilation capacity of the species, we measured the gas exchange of field plants at each population on multiple occasions (between 3 and 9 measurement dates) during the growing season between 2005 and 2010 (dates are provided in Table S1). All measurements were taken between 09:00 am and 15:00 pm. The number of plants sampled from each location was between 5 and 15. Fully expanded leaves, generally from the 6th to 12th node position from the shoot tip, were selected for measurements. For each leaf, a photosynthetic light response curve was taken using a photosynthesis system (LI‐6400; LI‐COR) coupled to a leaf chamber with a leaf area of 2 cm^2^ and an light‐emitting diode light source (6400‐40). Measurements were taken under ambient temperature, reference CO_2_ was set to 380 µmol mol^−1^ and vapor pressure deficit was kept within the range of 0.9–1.5 kPa. A light response curve was taken for each leaf, measurements started with photosynthetic photon flux density (PPFD) matching ambient levels and raised incrementally until light saturation to obtain maximum photosynthetic rate (A_*max*_), photosynthesis readings were recorded after rates have stabilized at each PPFD). PPFD was then returned to ambient level, confirming a return to initial rates before incrementally decreasing it to zero and dark respiration rate (Rd) was taken when rates stabilized. Relevant parameters were derived from the data using a curve‐fitting software (AQ Curve Analysis, Photosyn Assistant; Dundee Scientific).

For LMA determination and leaf nitrogen analysis, leaf samples from each location were collected, packed in ziplock bags and kept cool until measurements. These leaves included those used for gas exchange measurements as well as additional leaves from similar shoot positions of other plants at each site. We determined LMA from the dry mass of 8.5 mm diameter leaf disks, oven‐dried for 48 h at 70°C). Similar prepared dried leaf discs were used to determine leaf nitrogen content as % leaf dry mass using a nitrogen and carbon analyzer (Sumigraph NCH‐22F; SCAS). A calibration curve was made from an acetanilide standard (Wako Chemicals) to convert the spectrometer outputs into nitrogen mass.

### Estimating monthly carbon gain

**Figure 2:**
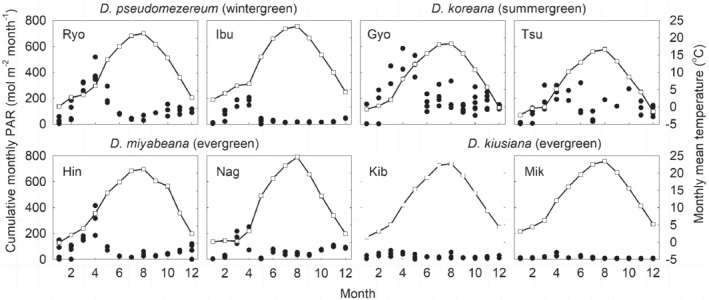
Cumulative monthly PAR (closed dots) and monthly mean air temperature (open dots) of the four *Daphne* species by two populations, based on long‐term (>2 years) microclimate data (at 2‐min intervals) collected from three replicated sites at each location. Location codes correspond to those given in Table 1.

To understand how potential carbon gain of Dpm might vary across the seasons, we estimated its carbon gain using actual variations in PAR and temperature from the Ibu site (Table 1 and Fig. 2) and plants raised in a common shaded garden at Ryukoku University (at 6.2% canopy openness, similar to those of Dpm habitats). Using five nearly mature (2–3‐year‐old) individuals, we first determined light‐ and temperature‐dependent photosynthetic responses in the laboratory. To do this, we measured each plant by placing it in a growth chamber (Conviron PGR15) set to one of six measurement temperatures: 5, 10, 15, 20, 25 and 30°C. A single fully expanded leaf, generally from the 6th to 10th node position from a shoot tip, was selected for measurements using LI‐6400 [at 380 ppm CO_2_, 50% relative humidity and one of the six preset chamber temperatures]. The leaf and LI‐6400 chamber assembly were enclosed in the growth chamber with measurement commands operated from outside the chamber. The plant was allowed 1 h to stabilize to each temperature before a light response curve was taken (with PAR at 0, 10, 30, 50, 100, 300. 500, 700, 900 and 1100 µmol m^−2^ s^−1^) at the set temperature. The procedure is repeated for the next temperature setting. By making the measurements in October using recently flushed autumn leaves, we could estimate peak physiological performance of Dpm. In deriving the photosynthetic response surface to temperature and PAR, we noticed that the response pattern under low light (i.e. 0–75 µmol m^−2^ s^−1^) was qualitatively different to that at higher light levels (75–1100 µmol m^−2^ s^−1^) and required different best fitting regression models as described below. The response surfaces composed of photosynthetic rates (Pn, *z*) generated from the six temperatures (Temp, *x*) x PAR (*y*) was fitted to 3D regression functions using SigmaPlot (Systat Software Inc.). The model that best fitted the low light response data was a paraboloid model of the following form:

(1) $$\begin{array}{cc} & \mathrm{Pn}=k–\left(a\;x\;\text{Temp}\right)+\left(b\;x\;\mathrm{PAR}\right)\\ & +\left(c\;x\;{\text{Temp}}^{2}\right)–\left(d\;x\;{\mathrm{PAR}}^{2}\right) \end{array}$$

where *k, a, b, c* and *d* are derived from the Paraboloid regression analysis. And the best fit model for the higher light response data was a Lorentzian model of the following form:

(2) $$\begin{array}{cc} & \mathrm{Pn}=a/\left(\left(1+{\left(\left(\text{Temp}‐k\right)/b\right)}^{2}\right)\right.\\ & \left.x\;\left(1+{\left(\left(\mathrm{PAR}‐l\right)/c\right)}^{2}\right)\right) \end{array}$$

where *a, k,b, l* and *c* are derived from the Lorentzian regression analysis.

The R^2^ value of the individual response surface ranged between 0.52 and 0.97 (median = 0.90 and 0.87 for Paraboloid and Lorentzian, respectively), and the anova (df = 4, 19), corrected for the mean of the observations, all showed significant values of *P* < 0.005. The model equations were then applied to actual microclimate data (from 10th to 15th day of the month) at 2‐min intervals from Ibu and Ryo (Table 1, Fig. 2). Carbon gain was summed over the 6 days and a mean daily carbon gain was calculated. Our models occasionally produced values that are inconsistent with the input (for example, a positive photosynthesis value when PAR = 0), in that case, we replaced it by extrapolating its likely value from the array of data adjacent to the error with similar input values. We also estimated the effect of leaf aging on the simulated carbon gain. This was done using both field gas exchange data and laboratory temperature response measurements to derive a correcting factor for leaf aging where a 5–10% monthly decline in photosynthetic performance was applied (detailed method is given in Supplemental Information).

### Statistical analysis

Means and differences in traits such as LMA, percent leaf nitrogen and gas exchange measurements among sites and species were determined using a nested two‐way anova (R Core Team 2013) with a sample size of between 5 and 15 per site. The difference between the regression slopes of A_*max*_ − Rd between Dpm and *D. koreana* was tested using a multiple regression with dummy variables:

(3) $$\mathrm{Rd}=b0+b1\;x\;A_{\max}+b2\;x\;d1+b3\;x\;A_{\max}\;x\;d1$$

where *b0, b1, b2* and *b3* are coefficients and *d1* is a dummy variable where Dpm = 0 and *D. koreana* = 1. The significance of the term A_*max*_ x *d1* tests the null hypothesis Ho: slope of Dpm = slope of *D. koreana*.

## Results

### Microclimate of the *Daphne*

The prevailing light environment of Dpm during summer was characterized by heavy forest canopy shade at canopy openness of 6.41 ± 1.56%, n = 17 for Ibu and 9.60 ± 1.81%, n = 28 for Ryo with ambient PAR at their lowest in July and early August, coinciding with their summer dormancy (Fig. 1). For example, at Ibu in August, we recorded PAR below the light compensation point (LCP) of 9.2 µmol m^−2^ s^−1^ in 89% of the time when low daytime irradiance and night time were combined, so if leaves were present at that time (estimated using simulated response data), this would lead to a respiration cost 6.2 times that of photosynthetic gain (i.e. when PAR is greater than LCP). Even for the brighter Ryo site, 60% of August PAR was less than LCP which would incur a cost of respiration nearly equaling (91%) that of the potential gain in carbon. It is important to note that concurrent with the deep shade, Dpm also experienced the highest temperature during these months (Fig. 2). Following leaf senescence of the deciduous canopy in autumn, canopy openness reached levels of about 30%. PAR sensors located near individual plants revealed that, while overall light levels were low during the winter months, some plants experienced periods of high PAR (>800 µmol m^−2^ s^−1^) while other plants may be temporarily snow‐covered (T. Lei, personal observation). Compared to Dpm sites, canopy openness at *D. koreana* sites on limestone outcrops were higher at 22.6 ± 4.2%, n = 29 (Gyo, refer to Table 1 for site description) and 32.4 ± 6.5%, n = 25 (Tsu) in mid‐summer. The light environment of the two evergreen species was generally dimmer, in particular that of *D. kiusiana*. The sugi‐hinoki forests (Kib and Nag sites, Table 1) cast a persistent shade on the order of 10% canopy openness. The mixed forest canopy of *D. miyabeana* provided an improvement in winter PAR similar to that in Dpm sites (Fig. 2). Soil moisture in the top 20 cm of soil largely remained above 10% by volume throughout the year but was lowest during the months of September and October (data not shown). Rainfall data for the region that includes the Dpm habitats of Ibu and Ryo (30‐year average for Hikone and Nagahama, Japan Meteorological Agency, http://www.data.jma.go.jp, last accessed Mar. 6, 2019) show a peak of 200 mm in July, declining to 100, 160 and 110 mm in August to October, respectively, indicating the absence of a clear dry season.

### Ecophysiological variations among *Daphne*

**Figure 3:**
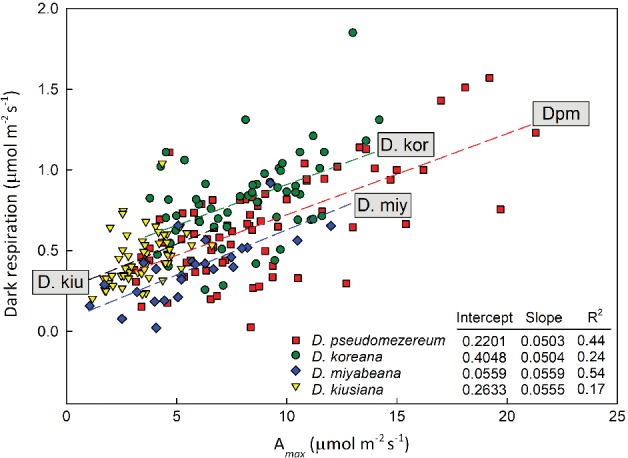
Photosynthetic properties of the four *Daphne* species native to Japan from multiple field measurements at each site. *Daphne pseudomezereum* (Dpm) exhibited a large range of gas exchange rates overlapping and exceeding *D. koreana,* despite its shadier habitat. Regression curve results are given in table with Dpm (red) and *D. koreana* (green) nearly identical in slope.

Dpm presented itself as a high‐light adapted plant through a number of attributes. First, in A_*max*_ vs dark respiration (Rd), Dpm showed a significant range of values that largely overlap those of *D. koreana* growing in bright upland habitats (Fig. 3). The regression fit for Dpm at Rd = 0.2201 + 0.0503 χ A_*max*_ is closely matched by that of *D. koreana* at Rd = 0.4048 + 0.0504 χ A_*max*_ (with virtually identical slopes, *P* = 0.99). The offset in the slopes is associated with a significant difference (Student's *t*‐test, *P* = 0.005) in Rd between *D. koreana* (0.816 ± 0.036 µmol m^−2^ s^−1^) and Dpm (0.667 ± 0.037 µmol m^−2^ s^−1^) suggesting a better carbon use efficiency in Dpm (Fig. 3). The two evergreen species (*D. miyabeana* and *D. kiusiana*) exhibited significantly lower means of A_*max*_ and Rd than the deciduous species (Table 2) but with similar regression slopes (Fig. 3). It is also clear that there were, on the whole, greater differences between sites for Dpm. Specifically, gas exchange of Dpm at Ibu was significantly lower than those in the Ryo population (Table 2A). This difference appears to be associated with the Ibu site being a more shaded location during the summer (Fig. 2). Secondly, there is a significant cohort by site variation in LMA. Between cohorts, the autumn (over‐wintering) leaves of Dpm can be as high as its evergreen congeners (Site Ryo: 55.8 g m^−2^) while the spring leaves tended to be low (31.1 g m^−2^) on the same order as the winter deciduous *D. koreana* (Table 2A). But at the Ibu site, the same difference between cohorts was not evident as LMA of both spring and autumn leaves were about 31 g m^−2^. Thirdly, consistent with other leaf‐level traits expressing high‐light‐adapted properties, Dpm leaves also have higher percent leaf nitrogen relative to its three congeners (Table 2).

**Table 2:** Key leaf traits of the four *Daphne* species native to Japan are shown in A. All measurements taken on recently matured leaves in early summer (*D. koreana, Daphne kiusiana* and *Daphne miyabeana*) or late autumn (*Daphne psuedomezereum*, after autumn leaf flush). Differences between species and sites for each trait are indicated by different letters following the mean values. Values are given as mean ± se. Results of differences between species and between sites within species of traits are shown in B. Data were analyzed using nested anova and Tukey HSD test.

(A)					
Species	Location code	LMA (g m^−2^)	%N	A_*max*_ (µmol m^−2^ s^−1^)	Dark respiration (µmol m^−2^ s^−1^)
*Daphne pseudomezereum* (Dpm)	Ibu	31.7 ± 0.9^c^	5.27 ± 0.24^a^	6.44 ± 0.42^c^	0.51 ± 0.04^c^
	Ryo	55.8 ± 4.0^a^	3.98 ± 0.10^b^	10.48 ± 0.65^a^	0.77 ± 0.05^ab^
*D. koreana* (kor)	Gyo	33.1 ± 2.4^c^	3.45 ± 0.26^bc^	7.10 ± 0.41^b^	0.74 ± 0.05^ab^
	Tsu	40.5 ± 4.1^bc^	2.94 ± 0.28^c^	9.11 ± 0.50^ab^	0.88 ± 0.05^a^
*Daphne kiusiana* (kiu)	Nag	52.9 ± 3.1^ab^	2.86 ± 0.16^c^	3.50 ± 0.23^d^	0.45 ± 0.03^c^
	Kib	56.5 ± 6.5^ab^	2.77 ± 0.27^c^	3.42 ± 0.22^d^	0.46 ± 0.03^c^
*Daphne miyabeana* (miy)	Hin	53.6 ± 2.3^ab^	2.85 ± 0.13^c^	5.44 ± 0.64^cd^	0.36 ± 0.05^c^
	Mik	58.2 ± 2.2^a^	ND	6.60 ± 0.82^bcd^	0.48 ± 0.05^bc^

(B)			
Trait	Species	Site (Species)	Diff. in Species means (*P* < 0.05)
LMA (g m^−2^)	***	**	kiu = miy > Dpm = kor
%N	***	**	Dpm > kor = kiu = miy
A_*max*_ (µmol m^−2^ s^−1^)	***	***	kor = Dpm > miy > kiu
Dark respiration (µmol m^−2^ s^−1^)	***	***	kor > Dpm > miy = kiu

### Simulated patterns of daily carbon gain

**Figure 4:**
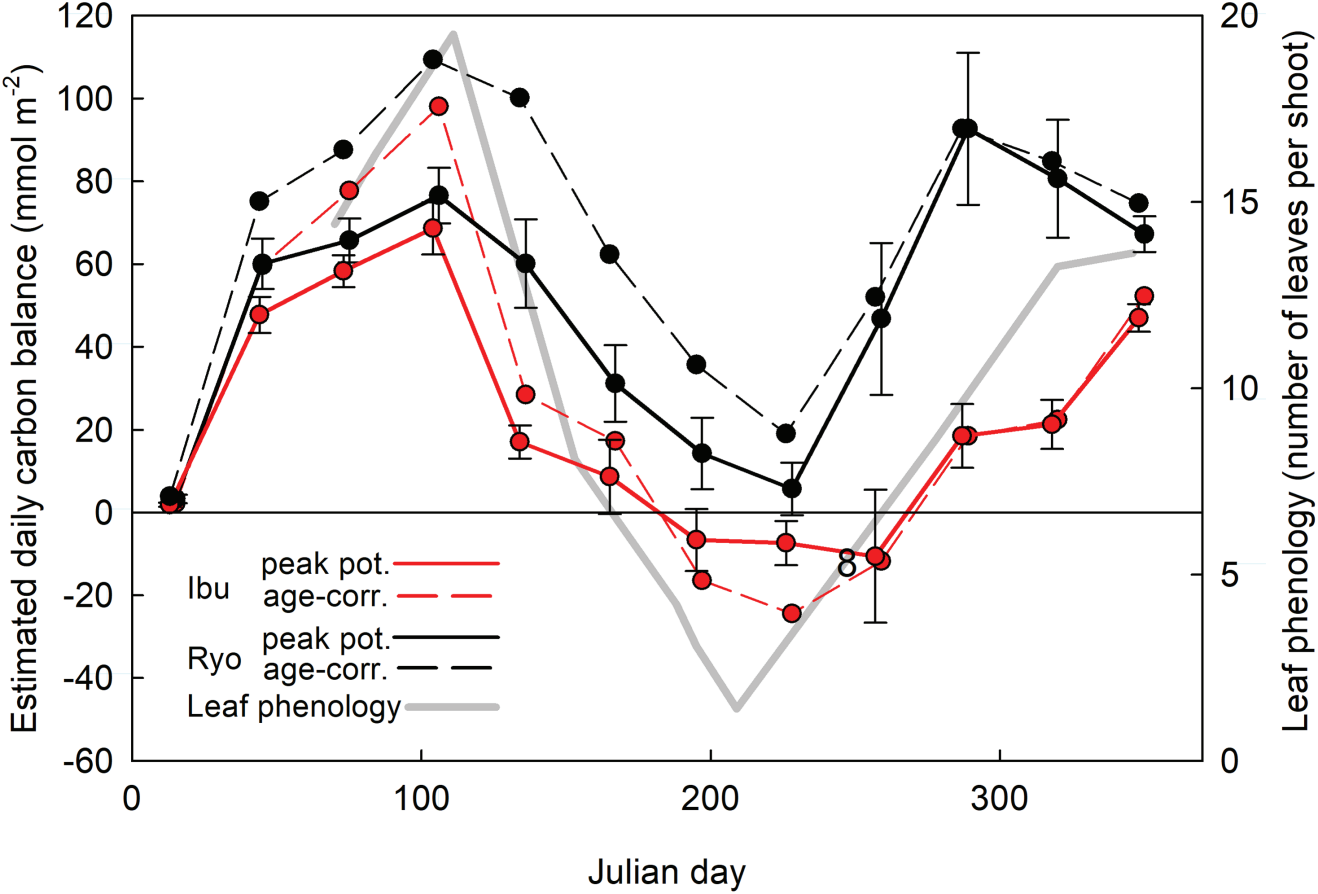
Simulation of leaf carbon gain in *Daphne psudomezereum* using photosynthetic response equations derived from newly matured leaves measured under different light and temperature settings. Response equations were applied to actual field microclimate data of Ryo and Ibu to derive mean daily carbon gain for each month (mean of five plants ± sd) accounted for the effect of leaf aging (solid lines, red for Ibu, black for Ryo). The dashed line for each site represents the response pattern not corrected for aging (error bars not shown). We also superimposed Dpm leaf phenology taken from a portion of Fig. 1 (gray line).

**Figure 5:**
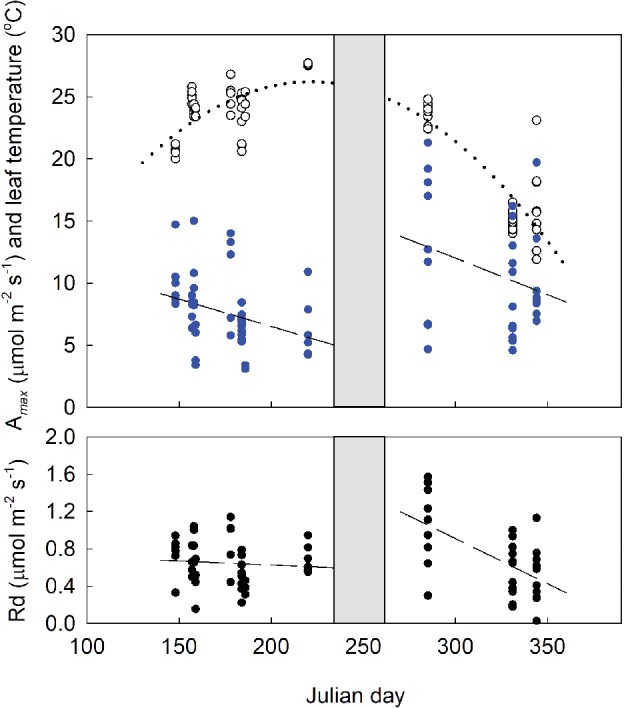
Field gas exchange measurements of Dpm showing leaf temperature (open circles) and A_*max*_ (blue dots) in the upper panel, and Rd (black dots) in the lower panel across season. Leaf temperature is shown with fitted equation (dotted line): Leaf temperature = −12.05 + 0.346 julian day (JD) – 0.0008 JD^2^ (R^2^ = 0.80). A_*max*_ and Rd were grouped as spring and autumn data on account of the midsummer dormancy (shown in gray). The regression lines (dashed) for each group are: A_*max*_ (spring) = 15.31–0.044 JD (R^2^ = 0.10); A_*max*_ (autumn) = 29.71–0.059 JD (R^2^ = 0.09); Rd (spring) = 0.711–0.0004 JD (R^2^ = 0.002); Rd (autumn) = 3.811–0.0097 JD (R^2^ = 0.35).

Initially, we used the same photosynthetic response data derived from newly matured leaves to estimate monthly carbon gain without accounting for leaf aging. This analysis allows us to ask what potential optimum carbon gain might Dpm achieve given the microclimate conditions where it grows. With this simulation, we found a pattern of daily carbon gain ranging between −25 and 109 mmol m^−2^ day^−1^ with increased carbon gains in autumn and spring and possible negative carbon balance in the summer (Fig. 4). Next, we estimated what effect leaf aging may have on the carbon gain pattern. We used field gas exchange measurements (Fig. 3) and estimated the likely rate of decline in photosynthesis and respiration, independent of seasonal changes in temperature (Fig. 5, Fig. S1). Based on the calculations, we estimated that gas exchange performance declined from 100% in October to 30% in the following August, applied the 5–10% a month decline to the simulated carbon gain (Fig. 4).

## Discussion

### Functional attributes of *D. pseudomezereum* (Dpm)

In the temperate forest of western Japan, Dpm shares the forest understory with summergreen species (including hardwood seedlings of *Acer* and *Quercus*), and evergreen species (such as *Aucuba japonica*, *Eurya japonica*, *Q* *uercus* *glauca* seedlings, and in some locations, *D. kiusiana*) that do not senesce in the summer. So, why is this plant behaving so differently from the others? Our results indicate that unlike the others, Dpm does not appear to possess the prerequisite shade‐adaptive traits for the forest understory as it shares many trait properties, such as LMA, A_*max*_ and leaf N, with *D. koreana,* its summergreen relative growing in much brighter locations (Figs 2 and 3; Table 2A,B). The LMA of Dpm showed a range of values depending on site and season but the autumn‐flushed leaves can attain values comparable to the evergreen *Daphne* and significantly greater than *D. koreana* (Table 2A, B). Leaves flushed in spring, and those grown in more shaded locations (such as Ibu), have lower LMA of 20–40 g m^−2^, a range more similar to other understory plants (Lei and Lechowicz 1998). The significant site differences in LMA also suggest that some Dpm populations, i.e. grown in Ibu, are expressing more shade tolerant attributes. Critical for Dpm is the production of a durable cohort of over‐winter leaves that sustains the necessary carbon gain through winter, even under relatively low air temperatures (Figs 2, 3, 4). The ability for Dpm to photosynthesize in low temperatures is evident in field measurements where A_*max*_ in December was 9.1 ± 0.9 µmol m^−2^ s^−1^ at air temperatures of 14.9 ± 0.6°C (Fig. 5). These early winter rates were comparable to those of *D. koreana* measured in mid‐summer (Table 2). Similar low temperature responses were seen in *D. jezoensis* grown in northern Japan where highest photosynthetic rates (ca. 6 µmol m^−2^ s^−1^) were obtained at 15°C, and even at 10°C, rates of 3–5 µmol m^−2^ s^−1^ were observed (N. Yamashita, unpublished). Percent leaf nitrogen (Table 2) is another trait that distinguished Dpm from typical shade‐adapted species (Lei and Lechowicz 1998), but despite the higher N content, Dpm does not appear to sustain substantial insect damage (T. Lei, personal observation). It is tantalizing to speculate that dormancy could also play a role in escaping predators in mid‐summer.

Dark respiration, together with LMA, A_*max*_ and leaf N, is considered an attribute of the ‘cardinal’ leaf design, linking the timing and duration of foliar presence to whole plant carbon gain (Reich et al. 1997, 1999, Wright et al. 2004, Kikuzawa and Lechowicz 2011). Leaf respiration received particular attention as a key trait in explaining leaf longevity because it appears to correspond well to a plant's shade tolerance (Wright et al. 2006, Baltzer and Thomas 2007). Using Baltzer and Thomas's data for tropical seedlings, Reich (2014) showed that leaves with higher dark respiration (Rd) not only demanded higher light environment for survival but are also associated with dramatically shorter leaf lifespans. We can infer from these results that for a shade plant, a lower Rd is critical for maintaining a lower whole plant LCP (Reich 2014) and those unable to do so, could face an unsustainable carbon deficit. Furthermore, studies have shown that shade tolerant plants can survive under low light not because they have higher photosynthetic rates or a better light capture efficiency than less tolerant plants, rather it is because they can maintain carbon gain by minimizing respiratory losses (Walters and Reich 2000, Craine and Reich 2005). Excessive respiratory cost has also been attributed as the driver for the initiation of dormancy in drought deciduous arid species (Mooney et al. 1975). These findings together suggest that, for Dpm, initiating summer dormancy at a time of high respiratory losses is a plausible response strategy. Under the combination of high temperature and low PAR, if Dpm plants had leaves in August, they would spend between 60 and 89% of the total time respiring and Rd would render daily carbon gain near 0 or even negative (Fig. 4). We also estimated that even a small difference in Rd could mean the difference between a summer month where carbon gain is positive (at Rd = 0.50 µmol m^−2^ s^−1^) or one with a carbon deficit (at Rd = 0.54 µmol m^−2^ s^−1^).

### Simulated patterns of seasonal carbon gain by Dpm

Our simulations clearly show that retaining leaves over winter contributes to achieving yearly net carbon gain in a typical Dpm environment (Fig. 4). It is also clear that such carbon balance is possible even if Dpm were to retain leaves over the summer, but it would be difficult without the boost in physiological performance initiated by the autumn flush. With or without accounting for leaf aging, the overall pattern did no differ qualitatively, with both showing a mid‐summer carbon deficit which was critically for Dpm, coincides with the period of summer dormancy in the field (Fig. 4). Simulation allowed us to visualize the summer carbon deficit, when in fact leaves are absent, and points to the stress Dpm would have to endure by spending most of the time in the dark and respiring at high rates (Fig. 5, Fig. S1). By sprouting its major cohort of leaves in autumn, Dpm is also unique among temperate woody species in deploying leaves that first experience a decreasing air temperature and daylength and then a reversal in spring prior to leaf shedding. Because of this seasonal effect, Dpm leaves first show an end‐of‐year decline in A_*max*_ when leaf aging is in sync with lowering temperatures and then continues to decline in the spring, but under rising temperatures. Newly sprouted spring leaves have a shorter lifespan where leaf carbon gain declines quickly into summer. Retaining leaves over winter offers Dpm a rare carbon gain opportunity under an open deciduous canopy that resembles evergreen saplings, where a large fraction of their lifetime carbon gain occurs during this time (Miyazawa and Kikuzawa 2005). The evergreen *D. miyabeana* also benefits from similar microclimate conditions and can, for example, amortize leaf costs sooner than *D. kiusiana*, growing mainly under a persistently evergreen canopy (Fig. 2). From this, we would expect a longer leaf lifespan for *D. kiusiana* than *D. miyabeana*, which is confirmed by our field phenology measurements where *D. kiusiana* has a mean leaf lifespan of 2.7–4.0 years, while that for *D. miyabeana* is 2.0 years (N. Yamashita, unpublished data). Although the summer dormancy of Dpm is unique in the temperate forest, the summer deciduous habit is common in arid regions as a response to extended drought (Mooney and Dunn 1970). Drought stress can also be exacerbated by shade, as the evergreen *Heteromeles arbutifolia* nearly ceases carbon gain in mid‐summer (Valladares and Pearcy 2002). Despite these similarities, Dpm and drought deciduous plants differ fundamentally in the primary drivers of their leaf response.

### Payback time and contribution to whole plant carbon gain

**Figure 6:**
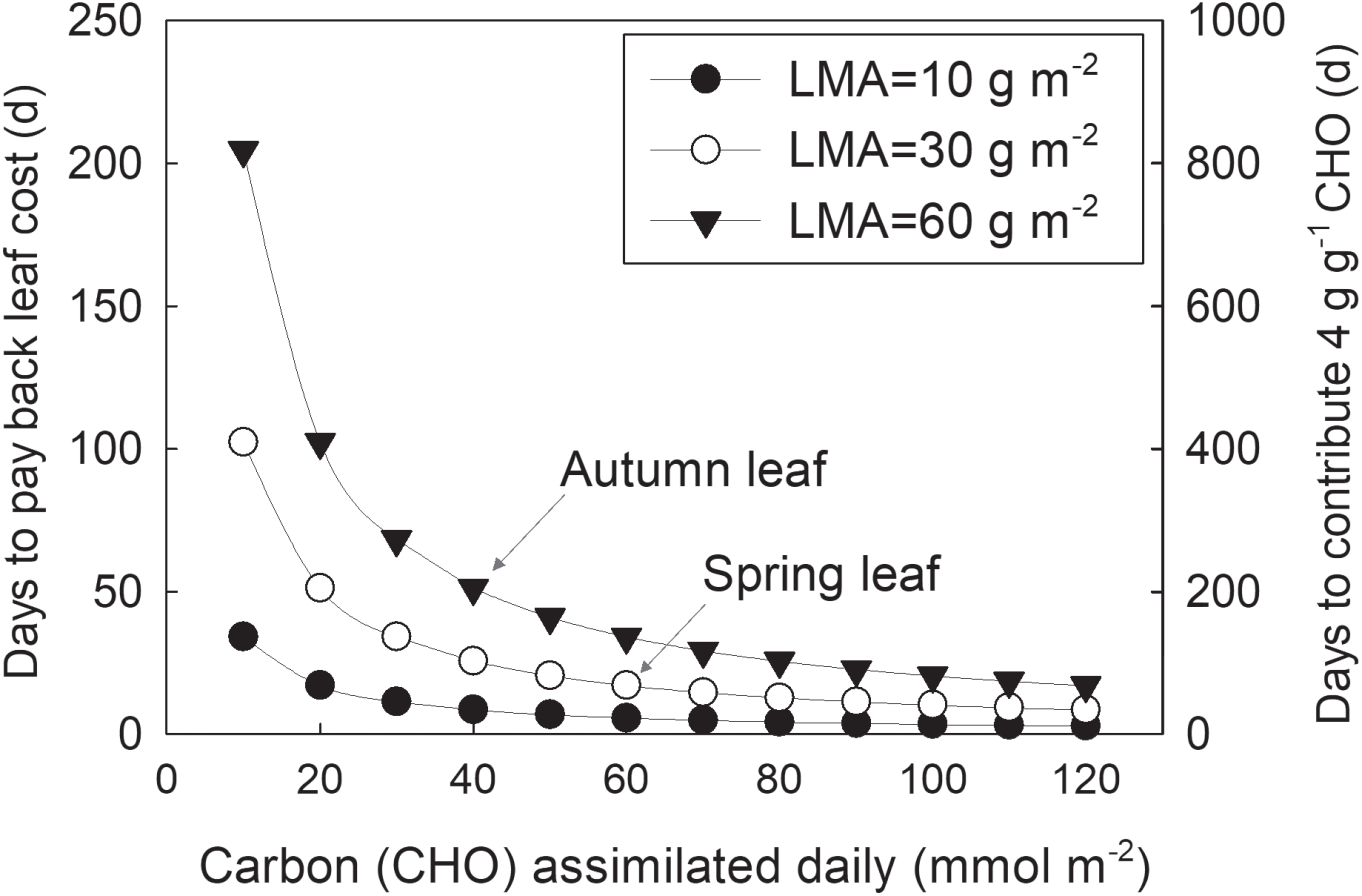
Estimated time (in days) to pay back leaf construction cost and the number of days required to contribute 4 g of carbohydrates (CHO) per g leaf CHO to the plant, as determined by mean daily carbon gain for leaves of different LMA (10, 30 and 60 g m^−2^ are shown). The same curves apply to both the payback time (left axis) and the time required to accrue additional 4 g of CHO per g leaf CHO (right axis). Calculations are derived from leaf morphology, microclimate data and results from the simulated carbon budget experiment shown in Fig. 4. The arrows point to values relevant to the discussion.

Relevant to the phenology of Dpm is whether summer dormancy is energetically compatible with the payback of its leaf cost and carbon return to the plant. We know that despite the large variation in leaf longevity and the diverse environments that leaves are exposed to, the cost of leaf construction is remarkably stable at approximately 1.5 g g^−1^ glucose (Williams et al. 1989, Villar and Merino 2001, Kikuzawa and Lechowicz 2011) and the lifetime carbon contribution by a leaf is consistently about 4 g g^−1^ (G‐net lifetime carbon gain in Kikuzawa and Lechowicz 2006). What this suggests, is that the longevity of a leaf may be determined by when the common set point in carbon gain per gram of leaf is reached. Given the wide range of leaf morphology, physiological performance and lifespan, the time needed to reach the set point would depend largely on the mean realized photosynthetic rate, leaf size and leaf tissue density. No leaf should live beyond a year, unless they are large, dense or are constrained by lower realized carbon gain (such as low PAR) that necessitates a longer payback time (Villar and Merino 2001). We estimated that deciduous leaves can repay their leaf construction cost in 46 days on average (range 13–112 days) and evergreen leaves in 219 days (range 92–427 days) using potential instantaneous photosynthesis rates (data provided in Kikuzawa and Lechowicz 2006) and assuming that these rates can be sustained for on average of 5 h per day. These payback times are consistent with earlier estimates of requiring 3 weeks under high light to 6 weeks in the shade for wild strawberry (Jurik and Chabot 1986). For Dpm, we can estimate the payback time based on the simulated carbon budget given in Fig. 6 at a range of LMA (serving as a proxy for leaf construction cost). Given that autumn leaves have an LMA in the range of 60 g m^−2^, and a daily carbon assimilation of 40 mmol m^−2^ (Fig. 4), it would require 51 days to payback the construction cost of such a leaf (Fig. 6), consistent with the deciduous averages above. Thus, even when we estimate the payback time using the actual daily carbon budget (and not just the mean A during daytime or A_*max*_), an autumn leaf can accrue carbon for 200 additional days during its lifespan, after paying back the leaf construction cost. This compares well with our estimate of the 204 days required to gain an additional 4 g g^−1^ (Fig. 6). Even a spring leaf with LMA of 30 g m^−2^ and a short lifespan of 60–80 days can payback its leaf construction cost in 17 days at 60 mmol m^−2^ (March, Fig. 4) and contribute close to 4 g g^−1^ to whole plant carbon gain with an additional 68 days. (Fig. 6). It is also clear that Dpm produces about three times more autumn leaves than spring leaves (T. Lei, unpublished data) suggesting that on a whole plant level, the leaf phenology deployed by Dpm is energetically viable in its habitat and consistent with the expected leaf functional longevity.

In conclusion, our findings support the view that Dpm is constrained by a suite of ecophysiological traits better suited to a higher light environment. But, by its ingenious flip in the timing of leaf deployment and leaf dormancy, it circumvented the fate of being a shade‐intolerant species (Craine and Reich 2005). For Dpm, the imperative of its summer dormancy and autumn flushing in maintaining carbon balance and enabling reproduction highlights is a novel adaptive response in a common selective landscape (Marks and Lechowicz 2006).

## Author contributions

T.L. conceived and designed the study and wrote the manuscript. T.L. and N.Y. jointly carried out the field study and data analysis. T.W. conducted the carbon gain simulation experiment. T.K. inferred the phylogeny of Daphne based on chloroplast and nuclear genomic analysis. T.M carried out the vegetation survey of the field sites.

## Supporting information


**Appendix S1**. Procedure for estimating leaf aging of Dpm.
**Table S1**. Dates of field gas exchange measurements.
**Fig. S1.** Temperature responses of A_*max*_ and Rd in pot‐grown Dpm.

**Fig**. **S2.** Dpm in snow taken in late February at Ibu. The naked terminal buds and flowers are visible.

**Fig**. **S3**. Dpm in winter, showing its growth preference for bases of trees and boulders.

**Fig**. **S4.** Dpm during summer dormancy (July 22). New leaf flush has appeared on the right shoot.

**Fig**. **S5.** The evergreen *Daphne kiusiana* with fruit, taken on June 11.

**Fig**. **S6**. *D. koreana* growing among limestone boulders, also preferred by Dpm, showing signs of leaf senescence (end of August).

**Fig**. **S7.** A typical Dpm habitat (Ryo) among limestones under a closed forest canopy in late June.
