# Supplementary material for: Why does Daphne pseudomezereum drop its leaves in the summer? An adaptive alternative to surviving forest shade
Source: Physiol Plant. 2019 May 7;168(1):77–87. doi: 10.1111/ppl.12972 (PMC7003882; doi:10.1111/ppl.12972)
Supplement: Supplementary file 1 — Appendix S1. Procedure for estimating leaf aging of Dpm. Table S1. Dates of field gas exchange measurements. Fig. S1. Temperature responses of Amax and Rd in pot‐grown Dpm. [file PPL-168-77-s001.pdf]

## Supporting information

**Table S1**. Dates of field gas exchange measurements at each *Daphne* site (site codes correspond to Table 1).

| Ryo | Ibu | Tsu | Gyo | Hin | Nag | Kib | Mik |
|---|---|---|---|---|---|---|---|
| 2005-07-03 | 2005/07/05 | 2005/07/10 | 2005/08/27 | 2008/07/31 | 2008/07/24 | 2008/07/30 | 2010/07/07 |
| 2006/06/27 | 2006/06/27 | 2006/08/02 | 2006/08/03 | 2008/06/24 | 2008/12/29 | 2008/12/11 | 2010/07/19 |
| 2006/10/12 | 2006/10/12 | 2007/07/05 | 2007/06/28 | 2010/07/27 | 2008/06/17 | 2008/06/18 | 2010/07/23 |
| 2007/06/07 | 2007/06/06 | 2007/08/06 | 2007/08/12 | 2010/07/28 | | | |
| 2007/12/10 | 2007/12/10 | 2008/09/14 | 2008/07/10 | 2010/07/29 | | | |
| 2008/08/07 | 2008/06/07 | 2008/07/15 | 2009/06/09 | 2010/07/30 | | | |
| 2008/07/02 | | | | | | | |
| 2008/11/26 | | | | | | | |
| 2008/05/27 | | | | | | | |

**Figure S1**. Temperature-dependent $A_{max}$ and Rd of common garden-grown Dpm plants (mean±sd, n= 5). Fitted curves for $A_{max}$ is $A_{max}$ = 0.556 *Temp – 0.011*$Temp^2$ ($R^2$ = 0.98) and for Rd is Rd = 0.63 – 0.05* Temp ($R^2$ = 0.80). Our regression for Rd did not include those of 5 and10°C because all Rd values at these temperatures were 0.

**Appendix 1. Procedure for estimating leaf aging in Dpm and applying the leaf aging correction**

We estimated the effect of leaf aging on gas exchange in the following manner. First, we see recently matured fall leaves declined towards year end in concert with a decreasing temperature (measured as leaf temperature, Fig. 5). As $A_{max}$ decreased from 13.7 μmol $m^{-2}$ $s^{-1}$ on JD280 to 9.5 μmol $m^{-2}$ $s^{-1}$ on JD 345, temperature dropped from 23°C to 15°C over the same period (75 days). This gives us a 30% decline in $A_{max}$ while temperature dropped 8°C (not accounting for aging). Next, we refer to our controlled environment measurements using potted plants (Fig. S1) where the same 8°C decrease saw a 21% decrease in $A_{max}$. So if we apply the temperature effect (not accounting for changes in temperature response due to leaf aging) to the field data, we obtained a 9% drop over 75 days or 3.6% per month. For Rd under the same temperature drop, we see a 0.5 to 0.05 μmol $m^{-2}$ $s^{-1}$ (90%) declined in controlled measurements where field data showed a more modest 1.1 to 0.5 μmol $m^{-2}$ $s^{-1}$ (45%) decrease, suggesting a larger effect from temperature than from aging. Similarly, spring field measurement of Rd in the field also showed no effect of aging under increasing temperatures (Fig. 5). Given these results, we will assume no effect of aging on Rd.

For $A_{max}$, there is a continued decline in spring and early summer as leaf (and air) temperatures rose. During this period, $A_{max}$ rose from 8.9 (JD 145) to 6 μmol $m^{-2}$ $s^{-1}$ (JD 220) resulting in a 32% decrease over 75 days, or 13% per month. Since temperature increased

from 22 to 27.5°C over the same period, we assumed that changes in $A_{max}$ was largely due to leaf aging.

Given the analysis above, we decided that a gradual decrease in gas exchange due to leaf aging in the following manner (beginning with pre-mature leaves in September at 90% our simulated values to 100% in October and then a 5% monthly decline through to March followed by a 10% monthly drop (see Table below). Since Rd has a large effect on carbon balance from June to August and because Rd does not show an age effect in our data (Fig. 5), we applied the age effect only to positive carbon gain values). We applied these rates to the simulated daily carbon balance values (shown in dashed lines in Fig. 4) and showed the age-corrected data in solid lines in the same figure.

| | Correction factor for leaf aging |
|---|---|
| Jan | 85% |
| Feb | 80% |
| Mar | 75% |
| Apr | 70% |
| May | 60% |
| June | 50% (applied to PAR>0 only) |
| July | 40% (applied to PAR>0 only) |
| Aug | 30% (applied to PAR>0 only) |
| Sep | 90% |
| Oct | 100% |
| Nov | 95% |
| Dec | 90% |
