# Supplementary figures and images for: Why does Daphne pseudomezereum drop its leaves in the summer? An adaptive alternative to surviving forest shade
Source: Physiol Plant. 2019 May 7;168(1):77–87. doi: 10.1111/ppl.12972 (PMC7003882; doi:10.1111/ppl.12972)

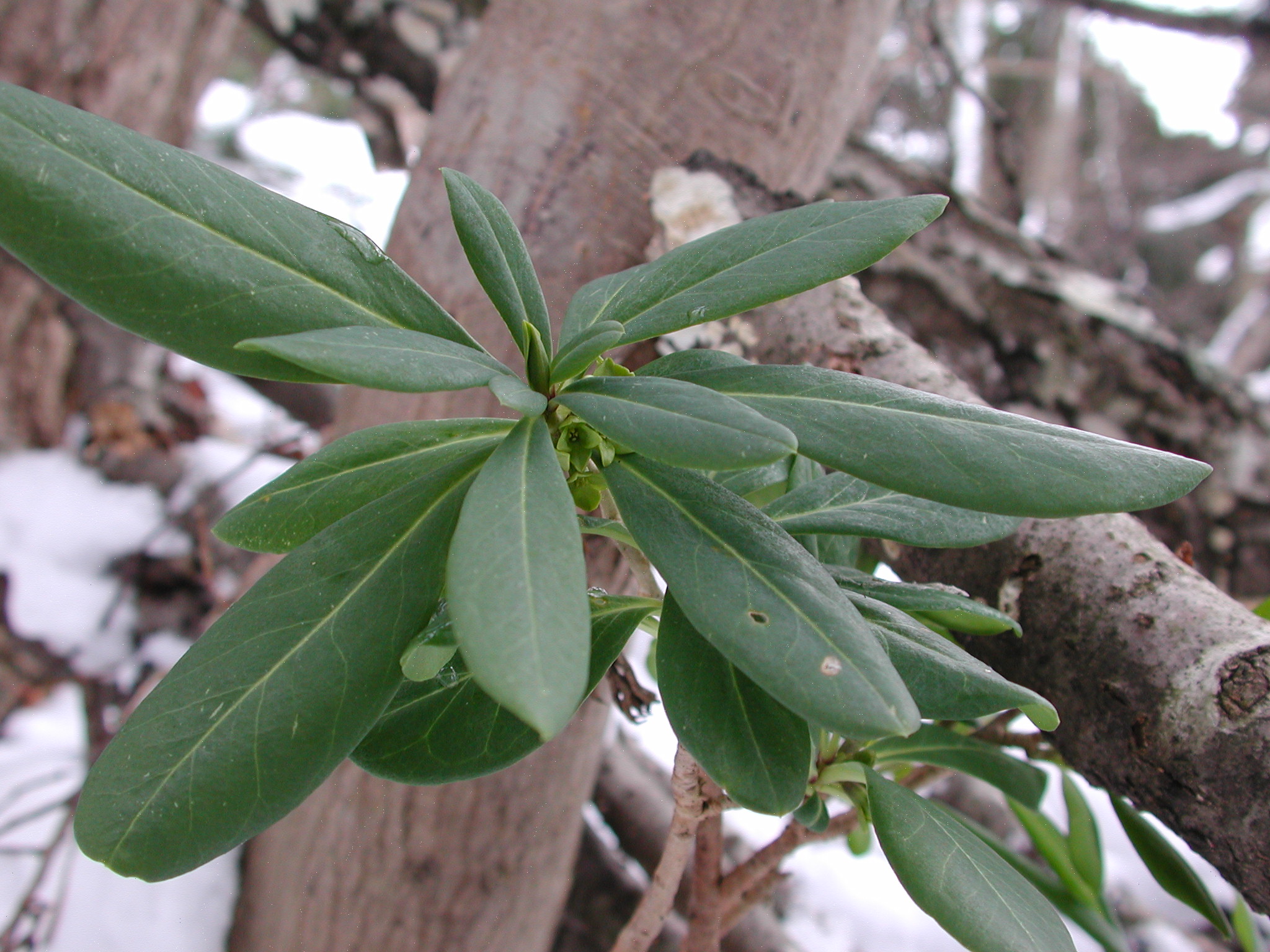

Supplement: Supplementary file 2 — Fig. S2. Dpm in snow taken in late February at Ibu. The naked terminal buds and flowers are visible. [file PPL-168-77-s002.JPG]

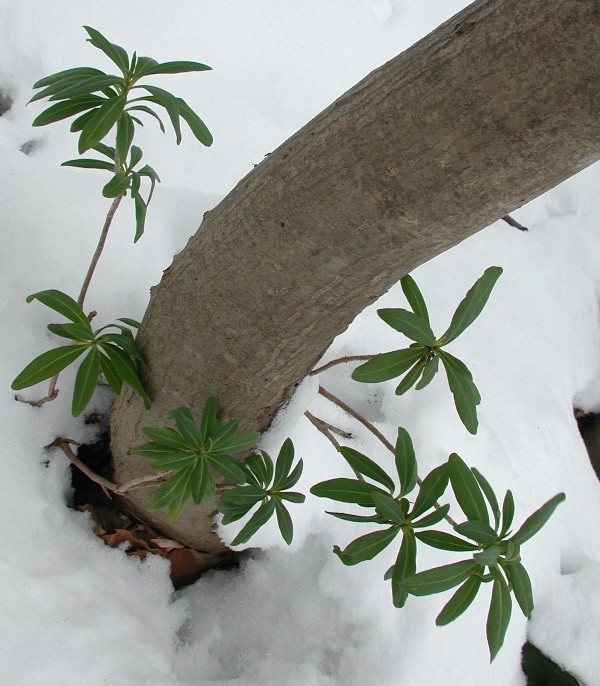

Supplement: Supplementary file 3 — Fig. S3. Dpm in winter, showing its growth preference for bases of trees and boulders. [file PPL-168-77-s003.jpg]

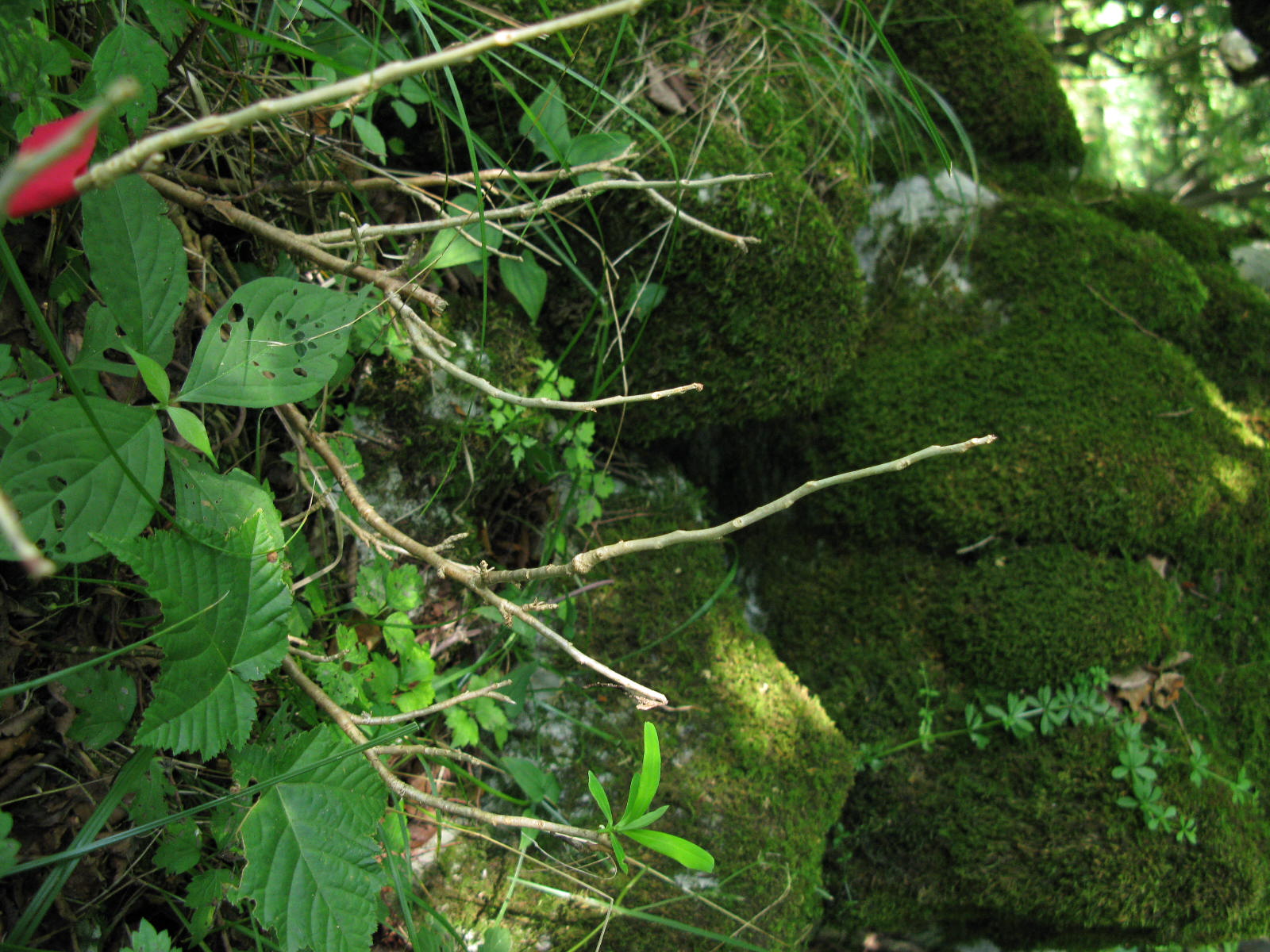

Supplement: Supplementary file 4 — Fig. S4. Dpm during summer dormancy (July 22). New leaf flush has appeared on the right shoot. [file PPL-168-77-s004.JPG]

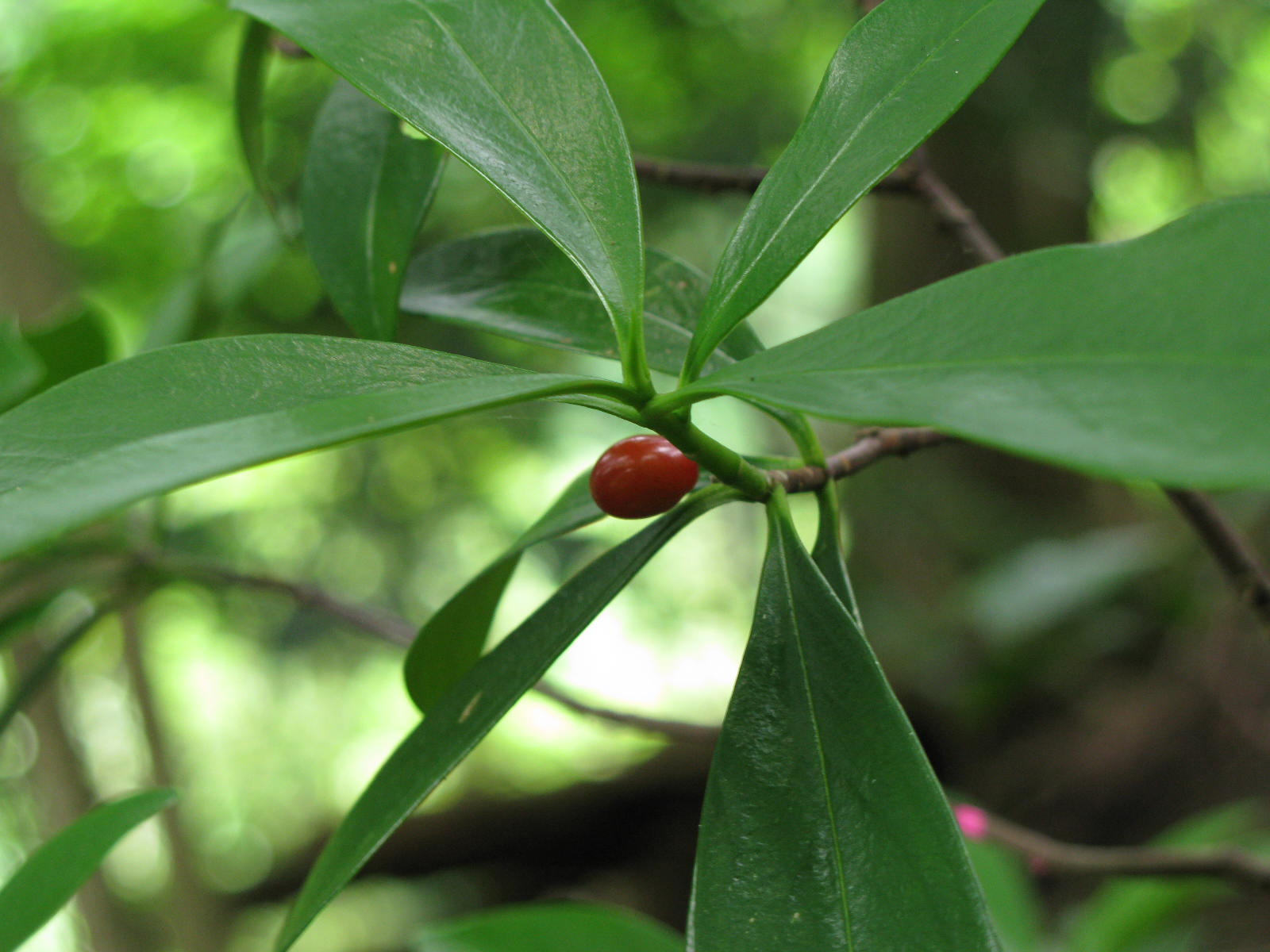

Supplement: Supplementary file 5 — Fig. S5. The evergreen Daphne kiusiana with fruit, taken on June 11. [file PPL-168-77-s005.JPG]

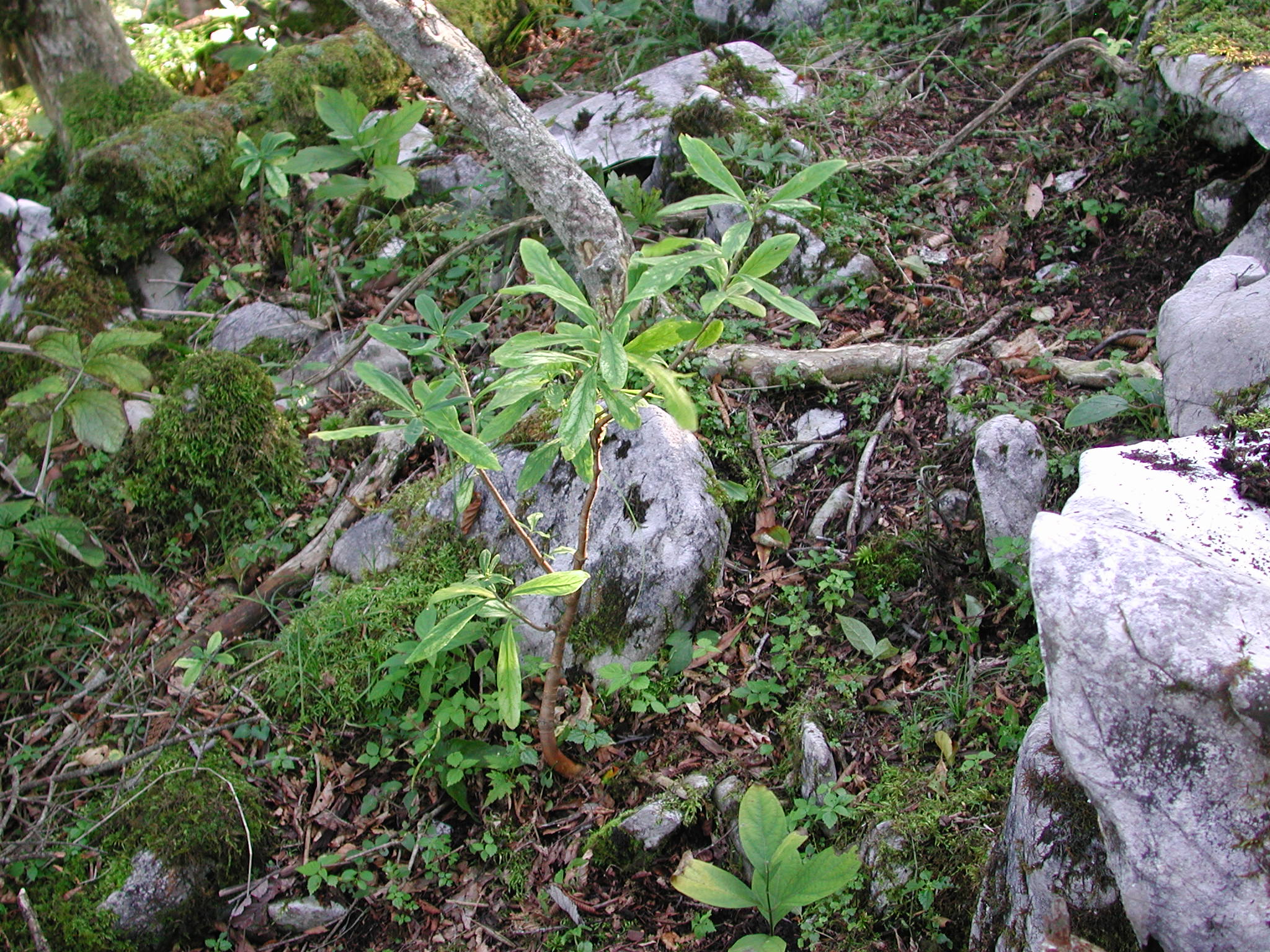

Supplement: Supplementary file 6 — Fig. S6. D. koreana growing among limestone boulders, also preferred by Dpm, showing signs of leaf senescence (end of August). [file PPL-168-77-s006.JPG]

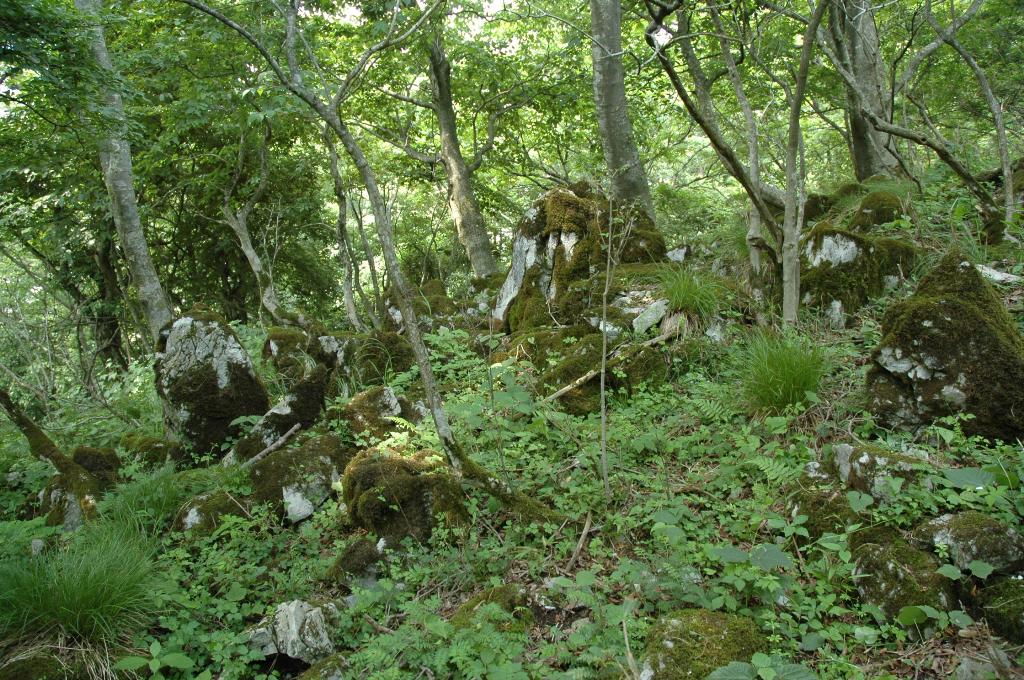

Supplement: Supplementary file 7 — Fig. S7. A typical Dpm habitat (Ryo) among limestones under a closed forest canopy in late June. [file PPL-168-77-s007.jpg]
